# Parietal theta burst TMS does not modulate bistable perception

**DOI:** 10.1093/nc/niae009

**Published:** 2024-03-17

**Authors:** Georg Schauer, Pablo Rodrigo Grassi, Alireza Gharabaghi, Andreas Bartels

**Affiliations:** Department of Psychology, University of Tübingen, Schleichstraße 4, Tübingen 72076, Germany; Centre for Integrative Neuroscience, Otfried-Müller-Straße 25, Tübingen 72076, Germany; Department for High-Field Magnetic Resonance, Max-Planck Institute for Biological Cybernetics, Max-Planck-Ring 11, Tübingen 72076, Germany; Department of Psychology, University of Tübingen, Schleichstraße 4, Tübingen 72076, Germany; Centre for Integrative Neuroscience, Otfried-Müller-Straße 25, Tübingen 72076, Germany; Department for High-Field Magnetic Resonance, Max-Planck Institute for Biological Cybernetics, Max-Planck-Ring 11, Tübingen 72076, Germany; Institute for Neuromodulation and Neurotechnology, University of Tübingen, Otfried-Müller-Straße 45, 72076, Germany; Department of Psychology, University of Tübingen, Schleichstraße 4, Tübingen 72076, Germany; Centre for Integrative Neuroscience, Otfried-Müller-Straße 25, Tübingen 72076, Germany; Department for High-Field Magnetic Resonance, Max-Planck Institute for Biological Cybernetics, Max-Planck-Ring 11, Tübingen 72076, Germany

**Keywords:** bistable perception, parietal cortex, TMS, theta-burst stimulation, multistability

## Abstract

The role of the parietal cortex in perceptual awareness and in resolving perceptual ambiguity is unsettled. Early influential transcranial magnetic stimulation studies have revealed differences in conscious perception following parietal stimulation, fuelling the notion that parietal cortex causally contributes to resolving perceptual ambiguity. However, central to this conclusion is the reliability of the method employed. Several prior studies have revealed opposing effects, such as shortening, lengthening, or no effect on multistable perceptual transitions following parietal stimulation. Here we addressed the reliability of continuous theta-burst stimulation (cTBS) on parietal cortex on the perception of bistable stimuli. We conducted three cTBS experiments that were matched to prior experiments in terms of stimuli, stimulation protocol, and target site, and used a higher number of participants. None of our cTBS experiments replicated prior cTBS results. The only experiment using individual functional localizers led to weak effects, while the two others led to null results. Individual variability of motor cortex cTBS did not predict parietal cTBS effects. In view of recent reports of highly variable cTBS effects over motor cortex, our results suggest that cTBS is particularly unreliable in modulating bistable perception when applied over parietal cortex.

## Introduction

When processing ambiguous information, our conscious perception may alternate spontaneously between mutually exclusive interpretations despite constant sensory input (Blake and Logothetis 2002). Ambiguous or multistable perception is hence a powerful approach to investigate the neural correlates of consciousness. However, previous studies of neuroanatomical locations involved in mediating perceptual transitions and conscious perception are still subject of an ongoing debate, not least because conflicting results have emerged (Tsuchiya et al. 2015, Koch et al. 2016, Brascamp et al. 2018, Block 2019).

A central dispute regards the question whether switches in conscious perception are initiated by higher-level cognitive frontoparietal areas (Leopold and Logothetis 1999, Sterzer et al. 2009, Zaretskaya et al. 2010, Panagiotaropoulos et al. 2012, Weilnhammer et al. 2021), or whether the resolution of ambiguity can be achieved in more posterior lower-level sensory cortices alone (Knapen et al. 2011, Frässle et al. 2014, Koch et al. 2016). Early neuroimaging reports showed that endogenously initiated perceptual switches were consistently associated with frontoparietal activity (Kleinschmidt et al. 1998, Lumer et al. 1998, Sterzer et al. 2002, Sterzer and Kleinschmidt 2007, Zaretskaya et al. 2010), while sensory areas reflected the content of perception (Tong et al. 1998). The switch-related activity in cognitive frontoparietal areas was interpreted as a potential cause of changes in conscious perception (Leopold and Logothetis 1999, Sterzer et al. 2009). However, subsequent imaging experiments suggested that activity in some (but not all—see Zaretskaya and Narinyan 2014) of the frontoparietal networks is instead a consequence of the transitions in consciousness, as their activity was reduced when using duration-matched ‘replay’ conditions (Knapen et al. 2011), when observers did not report switches (Frässle et al. 2014), or when unreportable or invisible displays were used (Brascamp et al. 2015, Zou et al. 2016).

More definite answers may hence be expected from causal stimulation techniques such as transcranial magnetic stimulation (TMS). One recent TMS study provided evidence for a causal role of prefrontal cortex in bistable selection employing a substantial number of participants (*n* = 30), with accompanying functional magnetic resonance imaging (fMRI) evidence suggesting a functionally similar computational role in a predictive coding framework of both parietal and prefrontal cortex (Weilnhammer et al. 2021). Unfortunately, prior TMS results on parietal cortex have been inconsistent. While several studies revealed modulations of perceptual fluctuations following stimulation of the parietal cortex (Carmel et al. 2010, Kanai et al. 2010, 2011, Zaretskaya et al. 2010, de Graaf et al. 2011), the direction or presence of these effects were inconsistent between studies: some reported shortening (Carmel et al. 2010, Kanai et al. 2011), others lengthening (Kanai et al. 2010, Zaretskaya et al. 2010), or no effect (de Graaf et al. 2011) (reviewed in Ngo et al. 2013, Brascamp et al. 2018). Moreover, some of these studies targeting the parietal cortex (Kanai et al. 2010, 2011, de Graaf et al. 2011) used the offline inhibitory protocol continuous theta-burst stimulation (cTBS) (Huang et al. 2005) that has recently been shown to have a relatively high rate of non-responders (>30%), a large inter- and intra-subject variability over the motor (Hamada et al. 2013, Corp et al. 2020) and prefrontal cortices and a poor reproducibility of the effects (McCalley et al. 2021, Ozdemir et al. 2021). In sum, while there is an agreement that parietal cortex contributes to steering perceptual alternations, there is uncertainty about its exact role and the reason for the inconsistency between causal studies.

One early but unresolved attempt for an answer was the proposal of a functional fractionation of the parietal cortex (Kanai et al. 2011) to explain incongruencies in TMS effects on bistable perception (Carmel et al. 2010, Kanai et al. 2010). The argument drew on a predictive coding framework, and was based on opposite correlations between grey matter density and the dynamics of bistable perceptions in posterior and anterior parietal regions: a posterior part of the superior parietal lobe (pSPL) was supposed to destabilize perception (Kanai et al. 2010), while an anterior part of the intraparietal sulcus (IPS) stabilized it (Carmel et al. 2010). The proposed fractionation within a predictive coding framework saw the pSPL as an area involved in generating prediction error signals and to increase thereby the probability of a perceptual switch, while the IPS was hypothesized to be involved in generating predictions (Kanai et al. 2011). Correspondingly, inhibitory stimulation of the pSPL using cTBS evoked a lengthening of percept durations (Kanai et al. 2010), while stimulation of the IPS using an inhibitory offline 1 Hz protocol (Carmel et al. 2010) and cTBS (Kanai et al. 2011) evoked a shortening of percept durations. However, the proposed fractionation could not account for all findings: another contemporaneous TMS study stimulating IPS just 3 mm away from the previous studies using an online 2 Hz protocol revealed opposite effects (a lengthening) and no effect after stimulation of the pSPL (Zaretskaya et al. 2010). Differences between protocols (1 Hz offline: Carmel et al. 2010, cTBS offline: Kanai et al. 2010, 2011 and 2 Hz online:, Zaretskaya et al. 2010), targeted areas (pSPL and IPS), and bistable paradigm used [binocular rivalry (BR): Carmel et al. 2010, Zaretskaya et al. 2010 and structure-from-motion (SFM):, Kanai et al. 2010, 2011] precluded a conclusive comparison between studies. Furthermore, the number of participants was generally low and varied considerably between these studies (Carmel et al. 2010: *n* = 6, Kanai et al. 2010: *n* = 10, 2011: *n* = 8, Zaretskaya et al. 2010: *n* = 15) (see Table 1).

**Table 1.** Overview of prior and current studies, their outcomes, and key parameters, sorted by the number of participants

Study	n	Target	Protocol	Localization	Effect
Exp. 1	20	IPS	cTBS	fMRI-based	Lengthening^a^
Exp. 3	19	IPS	cTBS	MNI-coords	Null
Exp. 2	15	IPS	cTBS	MNI-coords	Null
	15	pSPL	cTBS	MNI-coords	Null
Zaretskaya et al. 2010	15	IPS	2 Hz	fMRI-based	Lengthening
	15	pSPL	2 Hz	fMRI-based	Null
Vernet et al. 2015	14	IPS	Single pulse	MNI-coords	Shortening/null^b^
Kanai et al. 2010	10	pSPL	cTBS	MNI-coords	Lengthening
de Graaf et al. 2011	10	IPS	cTBS	MNI-coords	Null/lengthening^c^
Kanai et al. 2011	8	IPS	cTBS	MNI-coords	Shortening
Carmel et al. 2010	6	IPS	1 Hz	MNI-coords	Shortening^d^

Abbreviations: lengthening: lengthening of percept duration; shortening: shortening of percept duration; fMRI-based: use of individual functional MRI activations; MNI-coords: use of group-level, average coordinates in normalized space.

a Lengthening in frequentist statistics, but anecdotal evidence in Bayes factor analysis.

b Null result in two-sided test (appropriate given prior literature).

c Null result in passive viewing condition and a lengthening during cognitive control condition.

d No vertex control.

In the current study, we present a systematic investigation of cTBS effects on parietal cortex during bistable viewing. We present three highly matched experiments that examine the effects of parietal cTBS on the perception of bistable stimuli on a total of 41 unique participants. The first experiment aimed to replicate the shortening of percept duration following parietal IPS cTBS stimulation from Kanai et al. (2011), using the same SFM display and doubling the number of participants (*n* = 20). In the second experiment, we directly tested the proposed parietal fractionation (Kanai et al. 2011) by applying cTBS over the pSPL and the IPS using a BR display again using more participants (*n* = 15) than in the respective studies (Carmel et al. 2010, Kanai et al. 2010, 2011). We expected to see both, a lengthening (cf. Kanai et al. 2010, Carmel et al. 2010) (pSPL) and shortening (cf. Kanai et al. 2011) (IPS) of percept durations, respectively. In addition, because of the large inter-subject variability of cTBS over motor cortex (Hamada et al. 2013, Chung et al. 2016, Jannati et al. 2017, 2019, Corp et al. 2020), we investigated as an independent part of the second study how consistent cTBS effects over different areas are. We applied cTBS over the motor cortex and we tested whether subject-specific cTBS effects over the motor cortex were related to the cTBS effects on parietal cortex. In the third experiment, we tested the effect of IPS cTBS on three different bistable displays to examine both main effects and effect correlations of across participants (*n* = 19). Only the first of our three experiments led to a weak effect, while the other two delivered null findings. In addition, our second experiment revealed an absence of correlation between cTBS effects over parietal and motor cortex. We discuss our results in context of other studies considering target localization, power, and neural efficacy.

## Material and methods

### General rationale and overview

The three experiments, while independently conceived, were highly matched and therefore comparable: they targeted the same parietal area (IPS), used the same offline cTBS inhibitory stimulation protocol, vertex as a control condition, and measured the same outcome (differences in percept duration following cTBS stimulation).

The first experiment (Exp. 1), aimed to directly replicate the behavioural results of parietal IPS cTBS stimulation from Kanai et al. (2011), and thereby to answer the first question: are the effects of parietal cTBS on conscious perception consistent and replicable? Accordingly, we used the same SFM display as in Kanai et al. (2010) and (2011) to create bistability. Based on the previous report (Kanai et al. 2011), we expected to observe a shortening of percept duration following parietal IPS cTBS stimulation. The number of participants in Kanai et al. (2011) was 8, in the present study it was 20.

The second experiment (Exp. 2) investigated two questions: (A), whether the proposed fractionation of the parietal cortex as proposed by Kanai et al. (2011) can be replicated and, in view of incongruent results (B), whether differences in individual susceptibility to cTBS over motor cortex correlate with individual differences of behavioural cTBS effects over parietal cortex. The number of participants was 15.

In more detail on (A): the parietal fractionation proposed a perceptually destabilizing role of the posterior SPL (Kanai et al. 2010), and a stabilizing role of the more anterior part of the IPS (Carmel et al. 2010, Kanai et al. 2011). This fractionation was proposed to reflect distinct computational elements in context of predictive coding, and hence should generalize across bistable stimuli (as shown in Carmel et al. 2010). Accordingly, in Exp. 2 we ask: can we replicate the parietal cortex fractionation? We applied cTBS (as in Exp. 1 and Kanai et al. 2010, 2011) and used a BR stimulus (as in Carmel et al. 2010, Zaretskaya et al. 2010) instead of a SFM display. Kanai and colleagues also used cTBS and reported a lengthening of percept durations following pSPL stimulation (Kanai et al. 2010) and a shortening of percept durations following IPS stimulation (Kanai et al. 2011). Accordingly, we hypothesized to see the same qualitative patterns of modulation when using a BR stimulus.

In more detail on (B): although cTBS has been consistently shown to have an inhibitory effect (Wischnewski and Schutter 2015, Chung et al. 2016, Corp et al. 2020), there is a well-documented inter-subject variability in the effect of cTBS to motor-evoked potentials (MEP) following motor-cortex (M1) stimulation (Hamada et al. 2013, Chung et al. 2016, Jannati et al. 2017, 2019, Corp et al. 2020). Some of this inter-subject variability has been suggested to be explained by genetic polymorphisms (Cheeran et al. 2008, Jannati et al. 2017) or by which interneuron network is targeted via TMS (Hamada et al. 2013). In view of a lack of a direct assessment of the neural efficacy of parietal cTBS, we decided to indirectly assess it by performing a subsequent M1-cTBS experiment in the same participants of Exp. 2 (see Praß and de Haan 2019 for a similar rationale). Thus, in Exp. 2, we further ask: are behavioural individual differences in parietal cTBS correlated to the effects of M1-cTBS? We hypothesized that, if the effect of cTBS is dependent on subject-specific variables, differential MEP responses after M1-cTBS should correlate with the effects resulting from parietal cortex stimulation. Such a correlation would suggest that the effects of cTBS are generalizable across the cortex and would aid the interpretation of previous inconsistent results.

Finally, in Exp. 3, we tested whether the previously reported inconsistencies among results could be explained by differences in the bistable stimuli used: SFM (Kanai et al. 2010, 2011) versus BR (Carmel et al. 2010, Zaretskaya et al. 2010). Accordingly, in Exp. 3, we again targeted the right IPS using cTBS and tested its effects in three different bistable displays: SFM and two BR displays. This experiment was hence also a replication of the Exp. 1 and Exp. 2. In terms of analysis, it also allowed for a novel approach: the use of three different displays on the same participants allowed us to investigate the consistency of parietal cTBS effects between the three stimuli across the individuals. The number of participants was 19.

#### Participants

Prior to the three experiments, participants were screened to ensure the safety of TMS application (Rossi et al. 2009), psychophysically screened to ensure adequate bistable perception and median percept durations and asked to give written informed consent. All experiments were approved by the institute’s ethics committee and followed the Declaration of Helsinki. Participants had normal or corrected to normal vision. A total of 41 unique volunteers participated in the experiments. In Exp. 1, 2, and 3 we had a total of 20, 15, and 19 participants, respectively. Some participants took part in more than one experiment (in Exp. 1 and 2 = 2, in Exp. 2 and 3 = 3, in Exp. 1 and 3 = 4, and in all three experiments = 2).

#### TMS protocol and neuronavigation

##### Hardware

TMS pulses in all experiments were delivered using a figure-of-eight coil (MC-B70) connected to a MagPro X100 stimulator (MagVenture, Willich, Germany).

##### Resting motor threshold

Resting motor threshold (RMT) was individually defined for each participant during a screening session before the respective experiment. The RMT was determined visually by varying stimulation intensity over the left motor cortex until stimulation elicited a visible contralateral finger muscle twitch in ∼5 out of 10 pulses. Pulses were delivered holding the coil at a 45° angle relative to the sagittal midline with a frequency <0.3 Hz.

##### cTBS protocol

In all the three experiments, we used a continuous theta-burst protocol (Huang et al. 2005), consisting of bursts of three 50 Hz TMS pulses, applied every 200 ms for 47 s (600 pulses in total). This protocol is believed to induce cortical inhibition that has been shown to last for up to 50 min (Huang et al. 2005, Chung et al. 2016). However, the effect is strongest in the first minutes and consistent up to 30 min (Chung et al. 2016). Accordingly, all post-TMS behavioural measurements were planned to fall within this window of effect (<30 min after TMS stimulation). The TMS stimulation protocol was the same in all three experiments, but with different intensities (80% in Exp. 1 and 2 and 90% RMT in Exp. 3).

##### Target localization

**Figure 1.**
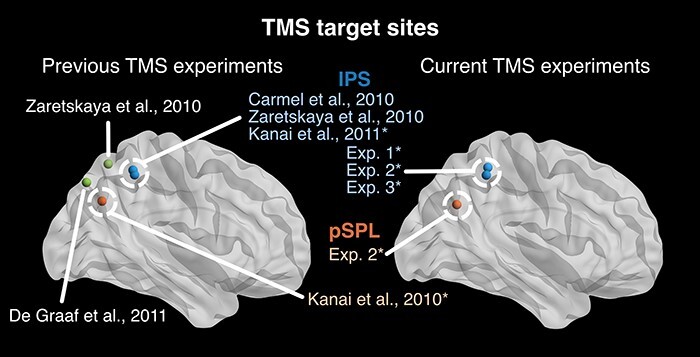
Overview of TMS target sites. Shown are parietal TMS target sites in comparable prior experiments using bistable displays (left) and the target sites used in the current study (right). TMS target visualization was done with BrainNet viewer (Xia et al. 2013). Asterisk: studies using continuous TBS

cTBS was applied in the three experiments to the right IPS and to the control site vertex on separate days. The right IPS was localized using individual fMRI measurements in Exp. 1 and using standard MNI coordinates in Exp. 2 and 3 (*x* = 36, *y* = −45, *z* = 51 from Lumer et al. 1998, also used in Kanai et al. 2011). Moreover, the pSPL was stimulated in Exp. 2 to test the proposed fractionation of the parietal cortex (*x* = 38, *y* = −64, *z* = 32, cf. Kanai et al. 2011) (see Fig. 1). For TMS stimulation, target coordinates were entered into the camera-based stereotactic neuronavigation system LOCALITE (Bonn, Germany) along with each participant’s structural T1 scan. During stimulation, the coil was held manually with its shaft pointing posterior–inferior at an angle of 45° to the floor. Moreover, we aimed to maintain a maximum distance between actual and ideal coil location of 1.5 mm at all times. The vertex location was localized based on externally visible anatomical landmarks (intersection between the nasion-inion and lateral midlines). For vertex stimulation the coil was held manually with its shaft pointed directly posterior, parallel to the floor.

Please note that the more posterior area of the parietal cortex stimulated in Exp. 2 (referred to as pSPL) is in fact located inferior to the superior parietal lobe and likely corresponds to an area of the inferior parietal lobule/angular gyrus instead. We nevertheless refer to this area as ‘pSPL’ to ensure consistency with prior literature as (Kanai et al. 2010, 2011) originally referred to this cluster as part of the SPL.

#### MRI scan acquisition

Structural MRI scans for neuronavigation were acquired using a 3 T Siemens Prisma using a 64-channel head coil (Siemens, Erlangen, Germany) at the Max Planck Institute for Biological Cybernetics, Tübingen. For each participant, we acquired a T1-weighted anatomical sequence (TR = 2000 ms, TE = 3.06 ms, TI = 1100 ms, FoV = 232 × 256 × 192 mm, voxel size = 1 × 1 × 1 mm, matrix 232 × 256, Flip angle 9°, 192 sagittal slices).

### Experiment 1

#### Overview

In Exp. 1, we intended to replicate the behavioural results following parietal cTBS stimulation from Kanai et al. (2011) using the same SFM display and measure concurrently neural activity using fMRI (results not shown here). Before the main experiment, participants underwent a screening experiment that examined behavioural parameters and established the RMT for TMS. The main experiment consisted of two or three stimulation sessions: fMRI data from the first session (control session, vertex-cTBS) was used to individually locate the parietal target site for the second session (IPS-cTBS). Half of the participants had a third session with vertex-cTBS to counterbalance the stimulation order. Below we describe the experimental paradigm (SFM), preliminary screening session, main experiment, and procedure to individually target localization.

#### Participants

A total of 20 subjects participated in the first experiment (mean age 23.6 years ± 2.9 SD; 14 females, 6 males, 17 right-handed) after screening 40 volunteers. During the screening, all participants were first checked for suitability for TMS using the criteria outlined by Rossi et al. (2009) and for psychophysical benchmarks appropriate for the fMRI experiment (see below). Half of the participants (*n* = 20) were then invited for the main experiment.

#### Experimental paradigm and setup

##### Structure from motion stimulus and task

**Figure 2.**
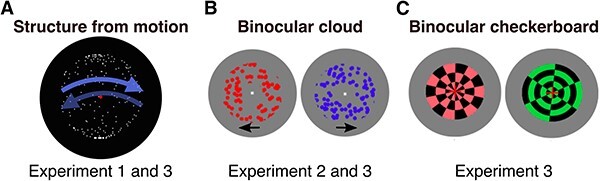
Visual stimuli used in the three experiments. (A) Structure from motion. Shown are 200 white dots on a black background moving coherently left and right to create the illusion of a rotating sphere. A red fixation dot is in the centre. Arrows indicate the possible perceived direction of movement. This stimulus was used in Exp. 1 and 3. Fusion aid used in Exp. 3 is not shown. (B) Binocular cloud stimulus. The display consists of 100 red or blue dots moving within a circular patch with a white fixation dot at the centre, presented separately to each eye. Within a given eye, 40% of the dots moved in the same direction, while the rest moved randomly. This binocular rivalry stimulus was used in Exp. 2 and 3. Fusion aid not shown. (C) Binocular checkerboard stimulus. This binocular rivalry stimulus was used in Exp. 3. Two checkerboards (green and red) were presented separately to each eye. Displays were flickering by alternating presentation of the checkerboard and its inverted image (where colours were exchanged with black) and rotating at 36°/s. Fusion aid not shown

We used the same SFM display previously used to create bistability (Kanai et al. 2010, 2011) (see Fig. 2a). This SFM stimulus is a bistable paradigm which induces the perception of a sphere rotating either to the left or to the right. It consisted of white dots moving horizontally back and forth within the boundary of a circle in a coherent fashion on a black background. The dots followed a sinusoidal velocity profile that was scaled such that it took every dot the same amount of time to move from one end to the other, and peak velocity was reached at the vertical axis of the stimulus. Dots moved once to either side and back to their starting position in 3 s. The sphere was 2° of visual angle in diameter and had a central red fixation dot (0.075° visual degrees).

Participants were instructed to press and hold one of the two buttons to indicate their current percept with their right hand. Participants pressed the left and right button during perceived left-wards or right-wards rotation, respectively. Moreover, participants were instructed to press no button when perception was unclear or mixed, which is rare for SFM stimuli.

##### Replay condition

In addition to the perceptually bistable condition, we also presented a replay condition. The replay condition was identical to the SFM stimulus, except for an added depth cue that exogenously induced percept switches. To create this 3D percept, the two SFM spheres were presented to each eye separately using a 3D shutter (DepthQ, Bellevue, USA) and polarization glasses. Dots from one sphere were presented slightly shifted along their motion trajectory to generate binocular disparity (individual dots had a maximum disparity of ca. 0.23° visual degrees). Thus, the SFM display was disambiguated, and the perceived rotation direction could be manipulated by adjusting which eye received the shifted image. The durations for the SFM replay condition were sampled from a gamma distribution of participant’s ambiguous SFM dominance durations recorded during the screening experiment.

##### Display setup

The stimuli were created and controlled using Psychtoolbox 3 (Brainard 1997) for Matlab (Mathworks, USA). In the screening experiment, they were presented on a monitor operating at 120 Hz (ASUS, Taiwan). Viewing distance was 700 mm. For the main experiment the stimuli were presented using a linearized projector operating at 120 Hz on a semi-transparent screen (29° × 16.5° visual degrees) using a mirror inside of the MR scanner. Viewing distance was 900 mm.

#### Screening and measurement of RMT

During the screening, participants were first checked for suitability for TMS using the criteria outlined by Rossi et al. (2009) and subsequently participated in a psychophysical test. They were shown the bistable SFM stimulus in 10 trials of 120 s. Participants were excluded from further testing if their median dominance duration was shorter than 4 s, longer than 8 s, or if predominance was either greater than 0.7 or smaller than 0.3. Predominance is defined as the cumulative duration of one percept (e.g. right-wards motion) divided by that of both percepts. The individual RMT was also measured during the screening. The mean RMT was 31.2% ± 3.97 SD of maximum stimulator output.

#### Experimental design and procedure

Each session of the main experiment took place on a separate day, and one of the TMS target sites (either vertex or IPS) was stimulated. Each session consisted of a total of five runs inside of the MR scanner: three prior to TMS and two following TMS. The first run (5 min) was to reacquaint participants with the stimulus, allow their percepts to stabilize and get them used to the scanner. Thereafter, participants completed two runs (each 9.2 min) of the main SFM test battery. The SFM test battery consisted of two 4 min blocks of SFM or replay condition interleaved with 24 s of fixation baseline (total of 9.2 min). The order of SFM and replay was constant within each participant but was counterbalanced across participants. Next, participants came out of the scanner and cTBS stimulation was applied. Immediately afterwards, participants re-entered the MR scanner and completed two runs of the SFM test battery.

Vertex stimulation occurred in the first session of each participant. This allowed us to use the fMRI data of the first session to functionally identify the individual IPS location involved in percept switches in each participant (see Section ‘Individual TMS site localization and fMRI ROI’). In the second session, IPS was stimulated. To exclude order and training effects, we invited half of the sample (*n* = 10) for a third session, during which vertex was stimulated again. For the later analysis, we only considered the second vertex appointment for these participants, such that across all 20 participants the order of stimulation site was counterbalanced (i.e. stimulation order in 10 participants was vertex–IPS, while it was vertex–IPS–vertex in the remaining 10 participants from which we used the last two sessions).

#### Individual TMS site localization and fMRI ROI

Images of the two pre-TMS fMRI runs of the first (vertex) session were used to define the IPS site as target for TMS stimulation for the second session. Data were analysed using the SPM12 package for Matlab (Welcome Trust Centre, Department for Neuroimaging, London, UK). Volumes were slice-time corrected, realigned, co-registered with the structural scan, normalized to MNI space and smoothed with a 12-mm full-width half-maximum Gaussian kernel. Using a standard general-linear model (GLM) approach, participants’ percept switches during the SFM and replay (i.e. onset times based on button presses) condition were modelled. We also included a block regressor for each condition (SFM and replay) separately, as well as six movement regressors and an orthogonal regressor of the mean signal intensity of each volume. The average peak MNI coordinates of the IPS region from the contrast *SFM block > replay block* were *x* = 30, *y* = −44, and *z* = 56. The Euclidian distances of this location to those published in prior studies were as follows: 7.8 mm compared to (Lumer et al. 1998), Kanai et al. (2011) and the coordinates used here in Exp. 2 and 3 (*x* = 36, *y* = −45, *z* = 51), 6.6 mm to Zaretskaya et al. (2010), and 7.9 mm to Zaretskaya et al. (2013). During the second session, we stimulated the individually defined IPS target locations (in native space) using a camera-based neuronavigation system.

To correlate brain responses with behavioural responses, we extracted beta estimates of the percept switches during viewing the bistable SFM from a region-of-interest centred at the right IPS region (*x* = 30, *y* = −44, and *z* = 56) including all significant voxels (at *P* < .005, uncorrected, from the *SFM block > replay block* contrast) within a sphere of 12 mm radius.

### Experiment 2 and motor cortex cTBS

#### Overview

In the second experiment, we stimulated the IPS as well as the pSPL to test their proposed differential roles in bistable perception (Kanai et al. 2011), along with vertex as control. We used a BR display comparable to those used in Carmel et al. (2010) and Zaretskaya et al. (2010). We further examined whether individual variability of parietal cTBS effects could be predicted by cTBS effects over the motor cortex, as determined by MEPs. As in Exp. 1, participants first underwent a screening experiment that examined behavioural parameters and measured their RMT. In the subsequent main experiment, participants took part in three sessions, each on a separate day, with one session for each of the three stimulation sites (IPS, pSPL, and vertex, counterbalanced across subjects). Finally, a last session was dedicated to motor cortex stimulation and MEP measurements.

#### Participants

For the second experiment, we recruited 34 volunteers. After screening (see below), a total of 15 participants took part in the main TMS experiment (mean age 23.93 years ± 2.74 SD; 13 females, 2 males, 12 right-handed). A total of 13 subjects also participated in the subsequent motor cortex cTBS experiment (mean age 23.85 years ± 2.76 SD; 11 females, 2 males, 10 right-handed).

#### Experimental paradigm and setup

##### Binocular ‘cloud’ rivalry stimulus

The binocular ‘cloud’ dot motion rivalry display we used was identical to one previously published (Brascamp et al. 2015), and described here again for completeness: the BR stimulus consisted of two apertures (inner radius = 0.45° visual degrees, outer radius = 1.25°) of randomly moving dots (radius = 0.08°; density = 165 dots per square degree; speed = 5.7 visual degrees per second) (see Fig. 2b). All dots in a given eye were either red-tinted or blue-tinted. Accordingly, the viewers’ perception spontaneously alternated between the perception of red dots and the perception of blue dots. To aid binocular fusion, the stimulus was surrounded by a white ring (radius = 2.9° visual degrees) that was surrounded by random white and black pixels within a square outline (of 7° visual degrees side length). At the centre of each aperture was a white fixation dot (white circular plateau with radius = 0.025° visual degrees, surrounded by a Gaussian radial falloff to background luminance, σ = 0.03° visual degrees). Each aperture’s dots had a within-eye motion coherence of 0.4, i.e. 40% of dots moved in a single direction (signal dots), while the rest moved randomly (noise dots). Dot motion direction was reset in 300 ms intervals, where identity and movement direction of all dots were randomly assigned, with the constraint that signal dots in one eye moved at a direction ± 90° (randomly chosen) relative to the direction of the other eye’s signal dots. The luminance of both colour tints was set to isoluminance using the heterochromatic flicker method.

Participants were asked to press and hold one of two buttons to indicate their current percept (right button during perception of the blue dots and the left button during the perception of the red dots). During periods of mixed perception, no button was pressed. All participants used their right hand for button presses. Importantly, in the standard BR condition participants were asked to ignore motion direction.

##### Display setup

The binocular stimulus was presented through a mirror stereoscope at a distance of 700 mm from the participant. A blackboard separator in the middle of the setup prevented participants from seeing anything apart from the intended image. All stimuli were displayed on a 27-inch monitor (Eizo, Japan) (at 60 Hz and 1600 × 1200 pixel resolution) controlled by a computer running Windows 7 using the Psychtoolbox 3 (Brainard 1997) package for Matlab R2014b (Mathworks, USA). There was no natural light contamination nor room lighting. A chin rest was used to minimize head movements.

#### Screening and measurement of RMT

During the screening participants performed 10 trials of 120 s of the binocular cloud rivalry stimulus. Participants were asked to report their currently occurring percept by pressing and holding down one of two buttons. Participants were excluded from further testing if their median dominance duration was shorter than 4 s and if eye dominance of either eye was >0.7. Please note that two further exclusion criteria based on acceptable gamma distributions and differences between conditions were also applied, but these conditions are not presented here (see below). The mean RMT measured during the screening experiment was 40.07% ± 4.08 SD of maximum stimulator output.

#### Experimental design and procedure

The experiment involved stimulation of three sites (IPS, pSPL, and vertex), applied during three different experimental conditions (BR, no-report BR, and invisible rivalry, cf. Brascamp et al. 2015). Note that for the purpose of the current manuscript we will only present results of TMS effects on the BR condition.

Participants were measured in three different sessions, each dedicated to one TMS stimulation site, each on a separate day. During each session, participants first completed an experimental run, followed by application of cTBS to either IPS, pSPL, or vertex (order was counterbalanced across participants). After TMS stimulation, participants completed another experimental run. Each experimental run consisted of nine trials (120 s each and three per condition).

In contrast to Exp. 1, stimulation over IPS was done using reported MNI coordinates (*x* = 36, *y* = −45, *z* = 51 from Lumer et al. 1998, cf. Kanai et al. 2011) and not individually defined targets. Stimulation over pSPL was done using the MNI coordinates *x* = 38, *y* = −64, *z* = 32 (from Kanai et al. 2010, cf., 2011). The coordinates in MNI space were projected onto the individual anatomical images and used for neuronavigation. Stimulation intensity was set to 80% RMT.

#### Motor-evoked-potential measurements

In view of inconsistent results following cTBS stimulation over the IPS and the pSPL (see ‘Results’ section below), we decided to perform a subsequent motor-cortex cTBS experiment on the same participants. The aim of this experiment was to investigate if the variability observed following parietal cTBS could be explained by subject-dependent factors as previously suggested (Cheeran et al. 2008, Hamada et al. 2013, Jannati et al. 2017, 2019). If indeed some of the inter-subject variability could be explained by subject-dependent factors, then this approach would allow us to indirectly assess the neural efficacy of parietal cTBS in the same participants (see Praß and de Haan 2019 for a similar rationale). Assuming that the inhibitory effect of cTBS can be generalized across the cortex and that the responses to cTBS are (more or less) consistent within a subject, we hypothesized that a correlation between the effect of motor-cortex M1-cTBS and the effects resulting from parietal cortex stimulation should be observable.

To test this hypothesis, we proceeded as follows. First, we remeasured the RMT in the participants using direct measurement of MEP amplitudes. RMT was identified by varying the stimulation intensity until MEP peak-to-peak amplitude reliably reached 50 µV in about 5 out of 10 consecutive pulses (Groppa et al. 2012). Then, a stimulus intensity that evoked a stable MEP with a peak-to-peak amplitude of 100 µV was determined. This stimulus intensity was used for the main MEP recordings. Thereafter, motor cortex excitability was measured by recording 30 MEPs with an inter stimulus interval between 4.5 and 5.5. s at stimulus intensity with the participant at rest. Following this baseline measurement, the cTBS protocol was applied to the motor cortex at 90% of the RMT defined in this session. Finally, after a rest period of 10 min, another 30 MEPs were recorded again. We compared the MEPs prior to cTBS and those 10 min after and correlated these differences to the differences in post-pre percent change observed after parietal cTBS.

The electromyography (EMG) was conducted using two Ag/AgCl AmbuNeuroline 720 wet gel surface electrodes (Ambu GmbH, Germany), which were fixated on the right extensor digitorum communis muscle at a distance of 2 cm. A third ground electrode was fixed to the elbow. The signal was filtered online between 0.16 Hz and 5 kHz. Electromyogram was recorded at 5 kHz through a BrainAmp ExG Amplifier (Brain products GmbH, Germany) and transferred to Matlab R2014a (Mathworks, USA) for online analysis and visualization as well as offline storage. TMS pulses were delivered using a figure-of-eight coil (MCF-B70) connected to a MagPro R30 + MagOption stimulator (MagVenture GmbH, Willich, Germany). Application was neuronavigated in the same manner as the main experiment.

Please note that, albeit some studies have suggested to categorize participants in cTBS ‘responders’ and ‘non-responders’ (Hamada et al. 2013, McAllister et al. 2013, Praß and de Haan 2019), new evidence from a large cooperative study (*n* = 430) reveals no such bimodal grouping (Corp et al. 2020). For completeness, we report here results from Pearson’s correlations between cTBS effects and also a separate analysis of the parietal cTBS effects on the M1-cTBS ‘responders’ (*n* = 10). We defined participants to be ‘responders’ if the direction of the mean post–pre percentage MEP differences were negative (below 0), thus suggesting an inhibitory effect of cTBS (Praß and de Haan 2019).

### Experiment 3

#### Overview

In Exp. 3, we addressed the question whether the inconsistent results in prior studies were due to differences in visual bistable paradigms (Carmel et al. 2010, Kanai et al. 2010, 2011, Zaretskaya et al. 2010). We compared the effect of cTBS stimulation over parietal cortex (IPS) (with vertex as control) using three different bistable paradigms: SFM, the binocular ‘cloud’ dot motion stimulus and a binocular checkerboard stimulus. This design allowed us to investigate the consistency of the parietal cTBS effects across different displays. Volunteers participated in two different sessions, one for each stimulation site (IPS or vertex, sequence counterbalanced across subjects). In the first session, the RMT was determined.

#### Participants

A total of 20 volunteers participated in the third experiment (mean age = 24.7 years ± 4.96 SD, 14 females, 6 males, 2 left-handed). Participants were screened for TMS safety prior to determination of RMT. The mean measured RMT of these participants was 30.6% ± 3.76 SD maximum stimulator output. Please note that one participant was subsequently excluded from the analysis due to missing data.

#### Experimental paradigm and setup

##### Bistable displays

Two of the stimuli (SFM and binocular cloud stimulus) were as described in Exp. 1 and Exp. 2, respectively. However, the SFM sphere was now 3° visual degrees in diameter and had a checkerboard fusion aid as described below. The binocular checkerboard stimulus (CKBD) consisted of two circular flickering checkerboards (see Fig. 2c). One was black and green while the other was black and red. The checkerboards had a diameter of 3.5° visual degrees and flickered at 7.2 Hz (red) and 9 Hz (green) respectively. The flicker was created through alternating presentation of the circular checkerboard and its inverted image (where colours were exchanged with black and vice versa). Moreover, the checkerboards rotated clockwise (36° per second). Around each checkerboard was a fusion aid, which was a black and white squared checkerboard frame with a side length of 7.7° and a central aperture of ca. 5°. The initial screen presented before the trial contained the fusion aid in addition to a central red fixation cross. The eye of presentation (i.e. which eye was presented with which checkerboard) was counterbalanced and determined randomly. The binocular stimuli used in Exp. 3 are shown side-by-side in the online supplementary video.

##### Display setup

The three stimuli were presented on a 27-inch monitor (ASUS, Taiwan) operating at 144 Hz, on a 50% grey background. Participants’ head position was fixed by a head- and chin-rest. There was no natural light contamination nor room lighting. Rivalry between two binocular stimuli (in the cloud and checkerboard paradigms) was created with a mirror stereoscope. An initial screen presented before the trials showed a fusion aid and a red fixation dot. The mirrors were carefully adjusted for each participant to achieve fusion of the fixation cross and lines. The distance between monitor and participant through the stereoscope was 700 mm. All stimuli were created and controlled by a stimulus computer (Ubuntu 17.10) running Psychtoolbox 3 (Brainard 1997) for Matlab R2014a (Mathworks, USA).

#### Experimental design and procedure

The third experiment was similar to the previous two: volunteers participated in two different sessions, in which either the IPS or vertex cTBS application occurred. In the first session also the RMT was determined. The order of the sessions was counterbalanced across participants. During each session, participants first completed an experimental run, followed by application of cTBS. Directly after cTBS application participants completed a second experimental run.

Each run consisted of six trials of 150 s of stimulus viewing (total of 15 min). In each run, the three stimuli appeared twice: for the binocular cloud and checkerboard stimuli once with red in the left and right eye, respectively. The display sequence was randomized.

Stimulation over IPS was done on the same reported MNI coordinates as in Exp. 2 (*x* = 36, *y* = −45, *z* = 51, from Lumer et al. 1998; cf. Kanai et al. 2011) and MR-guided neuronavigation. Please note that the stimulation intensity was increased to 90% RMT in this experiment.

### Behavioural data analysis

Behavioural responses during viewing of the bistable stimuli were analysed in the same way in all three experiments. Main measure of interest is the change of percept durations following cTBS over the parietal cortex (IPS and pSPL). This is measured by comparing the behavioural results post-TMS to pre-TMS for each experimental day (post–pre change) and comparing these to those collected during the control condition (vertex). Please note that the pre-TMS baseline was measured on each TMS testing day to control for day-dependent differences, such as arousal and attention.

Median percept durations of all percepts pre and post cTBS were extracted for each day (i.e. stimulation site) separately and used to calculate post-pre percent change, as (Median_post−_Median_pre_)/Median_pre_ × 100. Times in which participants pressed no buttons were excluded from the analysis. Normality of the data was assessed using the Shapiro–Wilk test. If applicable, differences in percent change were tested using two-sided paired *t*-tests and Bayes factor analysis (with a prior scale of 0.7071) (Rouder et al. 2009, 2012) in R (v4.1.2, R Core Team 2021). Otherwise, we performed non-parametric Wilcoxon signed rank tests and a Bayes factors for rank-based hypothesis testing (Van Doorn et al. 2020). Data from Exp. 3 were tested using a 2 × 3 repeated measures ANOVA with the factors TMS site (IPS, vertex) × stimulus type (SFM, CKBD, Cloud). Sphericity was assessed using the Mauchly test and corrected using the Greenhouse–Geisser method if necessary. Effect sizes were calculated using Cohens’ *d_z_* for paired designs (Cohen 1988) and partial eta squared (η^2^). All correlation coefficients were estimated using Pearson’s correlation.

### Overview of the experiments

All in all, we present here three TMS studies with highly matched experimental parameters and conditions (see Table 2). It is worth noting that all experiments were conducted by the same experimenters and measured in the same laboratory under close to identical conditions. Accordingly, we believe that observed differences can safely be attributed to experimentally designed parameters rather than lab-specific procedures.

**Table 2.** Overview of the parameters of the three experiments

Exp.	n	Target	Protocol	Intensity	Paradigm	Localization	Stimulator	Control
1	20	IPS	cTBS	80% RMT	SFM	fMRI-based	MagPro X100 (MC-B70)	Vertex
2	15	IPS, pSPL	cTBS	80% RMT	Cloud	MNI-coords	MagPro X100 (MC-B70)	Vertex
3	19	IPS	cTBS	90% RMT	SFM, Cloud, CKBD	MNI-coords	MagPro X100 (MC-B70)	Vertex

CKBD: binocular checkerboard stimulus; fMRI-based: localization based on individual functional MRI activations; MNI-coords: target using group level, average coordinates in normalized space.

Crucially, the three experiments were conducted separately, involving each time a largely different subset of participants and specific research questions. To test whether we measured enough participants, we estimated the expected power for each of the three conducted experiments. For this, we first extracted the mean and standard errors from plots of two published cTBS experiments over parietal cortex (Kanai et al. 2010, 2011) using WebPlotDigitizer (https://automeris.io/WebPlotDigitizer). Then, as the correlation between paired observations is necessary to calculate Cohen’s d_z_ in within-subject designs (Cohen’s d_z_ =$\left( {{m_x} - {m_y}} \right)/{\sigma _z},{\ }$with ${\sigma _z} = \sqrt {\left( {\sigma _x^2 + \sigma _y^2 - 2r{\sigma _x}{\sigma _y}} \right)} $), we used the smallest correlation between paired observations from our own results (*r* = 0.0067) to calculate the corresponding effect sizes as a conservative approach. Both cTBS experiments had large effects sizes of >0.8 (Kanai et al. 2010 = 0.95, 2011 = 1.15). Accordingly, using an effect size of 0.8, the expected power for the present experiments were 0.92 for Exp. 1, 0.82 for Exp. 2 and 0.91 for Exp. 3. with $\alpha $ = 0.05 and both tails. Moreover, our experiments had a similar viewing time per condition compared to previous cTBS experiments over parietal cortex (Kanai et al. 2010: two/three trials of 48 s, 2011: two/three trials of 4 min; Exp. 1: two trials of 4 min; Exp. 2: three runs of 2 min; Exp. 3: two trials of 150 s per stimulus and condition).

### Combined analysis

Additionally, we combined data from all three experiments to perform a pooled analysis using all 41 unique participants. We extracted the differences of percent change between IPS-cTBS and vertex-cTBS (IPS_post-pre %change_−vertex_post-pre %change_) and averaged these differences for all stimulus types in Exp. 3, as well as for those participants that took part in two (*n* = 9) or three experiments (*n* = 2) and tested for differences against zero. Data collected in the SPL-cTBS session from Exp. 2 were not included in this combined analysis.

Finally, to preclude averaging data from participants and to test for potential differences in the used stimuli, we modelled percent changes in a linear mixed model with TMS site (IPS and vertex) and stimulus (SFM, binocular checkerboard and binocular cloud) as fixed factors and participant as a random factor (using a maximum likelihood estimator) (Bates et al. 2015). Thereafter, we extracted the estimated marginal means of the difference in percent changes (IPS_post-pre%change−_vertex_post–pre%change_) separated per stimuli. Note that models without stimulus as fixed factor or including the experiment number as a random factor performed worse (i.e. had higher AIC values). As residuals were not normally distributed (based on Q–Q plots, Shapiro–Wilk test, and Kolmogorov–Smirnov test), we repeated the model after exclusion of three extreme values occurring after IPS stimulation when viewing the SFM stimulus (with 3 SD away from the group mean; two from Exp. 1 and one from Exp. 3).

## Results

### Experiment 1

**Figure 3.**
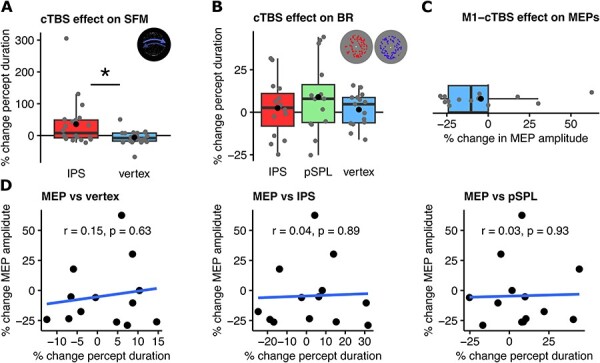
Effects of cTBS in Exp. 1 and Exp. 2. (A) Behavioural results from Exp. 1. Shown are changes in percept durations (post- versus pre-TMS) elicited through parietal IPS-cTBS during viewing of the bistable SFM display. *P < .05. Boxplots represent median, 25th and 75th percentiles (box) and ± 1.5 interquartile range (whiskers). Black dot represents the mean. (B) Behavioural results from Exp. 2 using the binocular cloud display and cTBS over two parietal sites. (C) MEP amplitude changes after cTBS stimulation over the primary motor cortex. (D) Correlations between changes in MEP amplitude after M1-cTBS over motor cortex and changes in percept duration following cTBS over vertex (left), IPS (middle) and pSPL (right)

The first experiment was a replication attempt of a prior study (Kanai et al. 2011). We tested for effects of cTBS to the right IPS (compared to vertex) on percept durations during viewing of a bistable SFM display. Our results are shown in Fig. 3a. In contrast to the previous study that stimulated the same site using the same stimulus (Kanai et al. 2011), we observed a lengthening of SFM percept durations following IPS-cTBS compared to vertex cTBS (Shapiro–Wilk test: W = 0.8, *P* = .0008; Wilcoxon signed rank test: V = 168, *P* = .017, non-parametric BF_10_ = 7.83). Please note that after exclusion of a participant that showed a difference over 3 standard deviations away from the group mean (increase of over 300% after IPS-cTBS), data was normally distributed (Shapiro–Wilk test: W = 0.96, *P* = .6) and showed similar results to non-parametric tests (*t*(18) = 2.5326, *P* = .021, Cohen’s d_z_ = 0.58, BF_10_ = 2.84). However, while the frequentist analysis still revealed a significant difference in percept durations following parietal stimulation, the Bayes factor analysis showed only anecdotal evidence (BF_10_ < 3) in favour of this difference.

Moreover, the effect of parietal cTBS compared to vertex cTBS on percept durations was not correlated to the effect of parietal cTBS compared to vertex cTBS on switch-related activity (i.e. post–pre differences in beta estimates) at the right IPS stimulation site (*r* = 0.04, *P* = .88).

### Experiment 2 and motor cortex cTBS

The second experiment re-examined the proposed differential roles of IPS and pSPL in bistable perception (Kanai et al. 2011). We applied cTBS over each of the sites (plus vertex) while measuring percept durations during binocular cloud rivalry. Results are shown in Fig. 3b. In contrast to the previous experiment, no significant effect in BR percept durations was observed neither following cTBS to the right IPS compared to vertex (*t*(14) = 0.2392, *P* = .8144, Cohen’s *d* = 0.0618, BF_10_ = 0.269), nor following pSPL stimulation compared to vertex (*t*(14) = 1.001, *P* = .3338, Cohen’s *d* = 0.2585, BF_10_ = 0.4032).

In view of these null results, we decided to perform a subsequent motor-cortex (M1) cTBS experiment on the same participants. The aim was to investigate if the individual variability observed following parietal cTBS could be explained by individual (subject-based) differences in the response to cTBS stimulation. First, we examined main effects of cTBS over M1 on MEP amplitudes. Across the group of participants a non-significant trend towards a decrease in MEP amplitude was observed (*t*(12) = −1.113, *P* = .2877, Cohen’s d = −0.3086, BF_10_ = 0.467, see Fig. 3c), which was marginally significant if an outlier showing >2 SD. from the groups mean percentage change was removed (*t*(11) = −2.311, *P* = .041, Cohen’s d = −0.667, BF_10_ = 1.923). Within individuals, comparisons between post- and pre-TMS revealed decreased mean MEP amplitudes for 10 of the 13 participants.

Hence, a trend towards the main inhibitory effect of cTBS could be reproduced over the motor cortex. However, this effect was not correlated to the differences in mean percept durations induced by cTBS stimulation in the preceding parietal cTBS sessions (Pearson’s correlation between changes in MEP amplitude, post- versus pre-cTBS, and changes in percept durations, post- versus pre-cTBS; IPS: *r* = 0.0411, *P* = .894; pSPL: *r* = 0.0265, *P* = .9316, vertex: *r* = 0.1478, *P* = .63, see Fig. 3d). In addition, we tested whether MEP amplitudes (post- versus pre-cTBS) were correlated with parietal versus vertex cTBS effects, but also here found no significant relationship (IPS-vertex: *r* = −0.0358, *P* = .9076; pSPL-vertex: *r* = −0.0276, *P* = .9286). Hence, there was no evidence for a relationship between effects of cTBS over the motor cortex and cTBS over the parietal cortex.

Finally, we performed a re-analysis of parietal effects, using only participants in whom the direction of the effect of motor cortex cTBS was negative (difference between post and pre percentage MEP amplitude following motor cortex cTBS < 0). Using this criterium, we defined 10 participants as ‘cTBS responders’. An analysis of parietal effects in these 10 ‘responders’ revealed no significant effect (compared against vertex: IPS: *t*(9) = 0.3159, *P* = .7592, Cohen’s *d* = 0.0999, BF_10_ = 0.3223; pSPL: *t*(9) = 0.4697, *P* = .6497, Cohen’s *d* = 0.1485, BF_10_ = 0.3393).

### Experiment 3

The third experiment aimed to test the replicability of parietal cTBS effects across three different bistable stimuli. The experiment was also a direct replication of Exp. 1 and 2 as cTBS was applied again to the right IPS (and vertex for control) while participants viewed the very same stimuli (SFM and binocular cloud rivalry), plus a further BR stimulus (checkerboards presented dichoptically). Crucially, by testing three different displays on the same participants we could correlate both the baseline responses, as well as the parietal cTBS effects on the different percept durations. Accordingly, this design allowed us to test the consistency of the parietal cTBS effects across a variety of bistable stimuli. It also allowed to test whether individual variability of cTBS effects was preserved across distinct stimuli.

**Figure 4.**
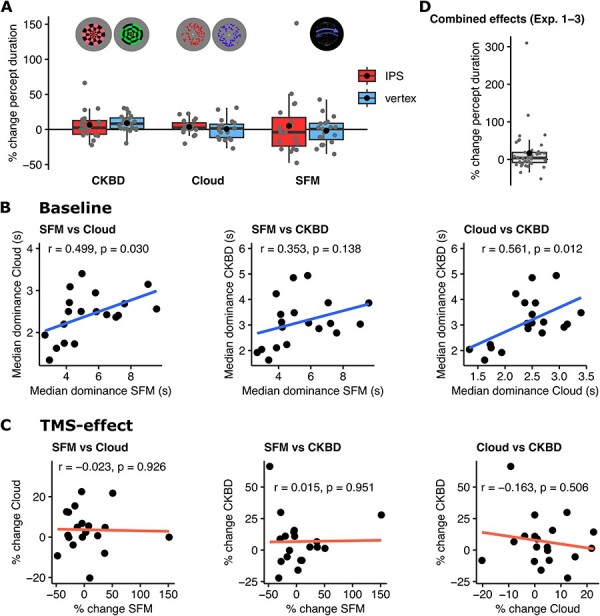
Effects of cTBS in Exp. 3 and combined analysis. (A) Behavioural results of Exp. 3. cTBS stimulation over the right parietal cortex (IPS) had no effect on the perception of three distinct bistable displays. Boxplots represent median, 25th and 75th percentiles (box) and ± 1.5 interquartile range (whiskers). Black dot represents the mean. (B) Correlations between baseline (pre-treatment) median percept durations of the parietal cTBS session of all bistable displays. (C) Correlations between percentage change of percept durations (post- versus pre-TMS) between the different bistable displays. CKBD: binocular checkerboard display. (D) Behavioural results from combined data from Exp. 1, 2, and 3 (*n* = 41). Overall, parietal cTBS had no significant effect on the perception of bistable displays

A 2 × 3 repeated measures ANOVA (TMS-site × stimulus type) was used to investigate effects on the percentage difference between pre- and post-TMS. The results are shown in Fig. 4a. We observed that there was neither a significant main effect of TMS-site (*F*(1,18) = 0.47, *P* = .503, partial η^2^ = 0.025), nor of stimulus type (*F*(2,36) = 0.75, *P*-corr = .453, partial η^2^ = 0.04, Greenhouse–Geisser ε = 0.795). Moreover, no significant interaction between the two factors was observed (*F*(2,36) = 0.43, *P*-corr = .593, partial η^2^ = 0.023, Greenhouse–Geisser ε = 0.732) (see Fig. 4a). Moreover, we conducted individual tests for each display separately. All results were non-significant, effect sizes negligible, and the Bayes factor analysis showed anecdotal evidence for the null hypothesis (SFM: Shapiro–Wilk test: W = 0.85, *P* = .007; Wilcoxon signed rank test: V = 104, *P* = .738, non-parametric BF_10_ = 0.25; binocular checkerboard: Shapiro–Wilk test: W = 0.86, *P* = .009; Wilcoxon signed rank test: V = 70, *P* = .332, non-parametric BF_10_ = 0.31; binocular cloud: *t*(18) = 0.819, *P* = .423, Cohen’s *d* = 0.1879, BF_10_ = 0.32).

Finally, while the median baseline percept durations between stimuli in the parietal cTBS session were mostly positively correlated (see Fig. 4b; Pearson’s correlation between SFM and binocular cloud: *r* = 0.499, *P* = .0297; SFM and binocular checkerboards: *r* = 0.353, *P* = .138; binocular cloud and binocular checkerboards: *r* = 0.561, *P* = .012), there were no such positive correlations between parietal cTBS *effects* (i.e. percent change of percept durations post- versus pre-TMS) between stimuli (see Fig. 4c; Pearson’s correlation between SFM and binocular cloud: *r* = −0.023, *P* = .926; SFM and binocular checkerboard: *r* = 0.0152, *P* = .951; binocular cloud and binocular checkerboard: *r* = −0.163, *P* = .5056).

### Combination of Exp. 1, 2, and 3

In a further analysis, we combined the (averaged) differences of percent change between IPS-cTBS and vertex-cTBS from all 41 unique participants from all three experiments. After extraction of two outliers with IPS-vertex differences of >100% (one 309.7% and the other 117.4%, both from Exp. 1), data revealed no significant difference, weak effects, and anecdotal evidence for the null hypothesis (see Fig. 4d, Shapiro–Wilk test: W = 0.955, *P* = .125; *t*(38) = 1.7, *P* = .0973, Cohen’s *d*_z_ = 0.27, BF_10_ = 0.64, mean difference ± SD was 6.85% ± 25.15; 95% CI [−1.31, 15]). Yet, even with inclusion of the outliers, the median difference in percent change between parietal and vertex cTBS stimulation was 3.99 (25th percentile = −7.93, 75th percentile = 18.79). This small difference was not significantly different from 0 and the non-parametric Bayes factor analysis reveals no evidence for it (Shapiro–Wilk test: W = 0.61, *P* < .001; Wilcoxon signed rank test: V = 567, *P* = .0779; non-parametric BF_10_ = 1.48).

Finally, a linear mixed model revealed an interaction between TMS site (IPS-vertex) and stimulus (SFM, binocular checkerboard and binocular cloud), driven by a lengthening of percept duration following IPS stimulation when viewing the SFM stimulus (in Exp. 1 and Exp. 3). The estimated marginals mean of the contrast IPS-vertex (mean and SE) were 24.75% ± 7.07, −2.21% ± 10.12 and 2.08% ± 7.57, for SFM, binocular checkerboard and binocular cloud displays, respectively. However, after exclusion of three outlier IPS values (3 SD away from the residual’s mean, two from Exp. 1 and one from Exp. 3, all from SFM trials), residuals were normally distributed (Kolmogorov–Smirnov test, *P* = 0.198) and the corresponding model revealed only a weak difference in estimated means when viewing the SFM stimulus (the estimated lengthening was now 10.17% ± 4.67). Accordingly, the results from the linear mixed models reflect a small difference of effects between stimuli, driven by the significant lengthening of percept duration while viewing SFM, as expected by the lengthening results from Exp. 1.

## Discussion

### Overview

Our first experiment (*n* = 20) explored the effect of anterior IPS cTBS stimulation on the perception of a bistable SFM display. Consistent with a previous 2 Hz online TMS study (Zaretskaya et al. 2010) (*n* = 15) we found a weak, yet significant lengthening of percept durations. Both of these studies used individual functional localizers for TMS target localization. Note that these results are inconsistent with a previous 1 Hz TMS study (Carmel et al. 2010) (*n* = 6) and a SFM cTBS study (Kanai et al. 2011) (*n* = 8) that both reported shortenings of percept durations. These studies as well as our Exp. 2 and 3 used average coordinates for TMS site localization. In the second experiment we aimed to replicate the previously proposed functional fractionation of the parietal cortex (Kanai et al. 2011), namely a lengthening of percept duration following pSPL cTBS and a shortening following IPS cTBS (in contrast to the results from our Exp. 1). We used a BR stimulus (as in Carmel et al. 2010, Zaretskaya et al. 2010), included the stimulation of a further parietal area (pSPL) (as in Kanai et al. 2010) and also investigated in a subsequent motor-cortex cTBS experiment if variance in the results could be explained by subject-dependent responses to cTBS. In contrast to previous studies and to Exp. 1, we observed no behavioural effects and there was no relation between effects of parietal cTBS and changes in MEP amplitude after motor cortex cTBS. Finally, in a third experiment, we investigated if the divergent results could arise from differences in visual stimulation and tested the effect of cTBS over IPS using three different bistable displays. Again, we found no behavioural effects and therefore did not replicate the previously observed behavioural effects following parietal stimulation (Carmel et al. 2010, Kanai et al. 2010, 2011, Zaretskaya et al. 2010), nor the results from Exp. 1.

In sum, the weak result in Exp. 1 (*P* = .02, Bayes factor analysis: anecdotal evidence) and null results in Exp. 2 and 3 (with some anecdotal evidence for the null hypothesis by a Bayes factor analysis), lead us to conclude that cTBS over parietal cortex does not consistently affect the dynamics of bistable perception (or does so in a weak and poorly replicable manner, see Exp. 1 and cf. de Graaf et al. 2011). Crucially, we conducted the experiments in near-to-identical conditions compared to each of the corresponding prior experiments. We hence assume that the higher statistical power in our experiments (>0.8 in all experiments) to detect an underlying effect was the key difference compared to prior experiments.

We further show that this ineffective modulation is true for two central areas targeted within the parietal cortex (Exp. 2) and generalizes across a battery of distinct bistable stimuli used (Exp. 3). Finally, while the linear mixed models using data from all experiments suggest a difference of parietal cTBS effects based on stimulus types, this difference was driven by the lengthening results from Exp. 1 and a few outliers viewing the SFM stimulus. Still, these results open the intriguing—albeit speculative—interpretation that parietal cTBS effects were only observed during viewing of the SFM stimuli, potentially because of the parietal role in extracting 3D structure from motion (e.g. Vanduffel et al. 2002, Murray et al. 2003) and in conscious perception of structured stimuli (Zaretskaya et al. 2013, Grassi et al. 2016b, 2018), rather than in resolving perceptual ambiguity.

The question that remains is *why* cTBS over the parietal cortex is ineffective in consistently modulating bistable perception. Is the parietal cortex reliably activated during perceptual switches? Do our three cTBS studies present reliable evidence that the parietal cortex is not causally involved in bistable perception? Or are the weak/null results rather evidence of methodological problems, such as insufficient power or the high variability/ineffectiveness of cTBS?

To correctly interpret our empirical null results we need to consider the following three elements (de Graaf and Sack 2011, 2018): (i) Did we stimulate the right areas? (*Target localization*), (ii) Did we have enough power to detect an effect? (*Power*), and (iii) Did we use a method that effectively disrupted parietal functioning? (*Neural efficacy*)

### Target localization

Are the inconsistencies due to targeting the wrong areas or differences in localization methods? In terms of the targeted area all experiments targeted the very same IPS area as previous reports (Carmel et al. 2010, Zaretskaya et al. 2010, Kanai et al. 2011), either based on individual fMRI responses (Exp. 1, distance for previous fMRI reports  < 1 cm, and Zaretskaya et al. 2010) or using the same, previously reported coordinates (Lumer et al. 1998), such as (Carmel et al. 2010, Kanai et al. 2011), deeming their comparison reasonable and precise (see Fig. 1).

It is worth noting that the only significant effect of our studies occurred in the one experiment using individually defined target sites based on fMRI results (Exp. 1). The observed lengthening of bistable percept durations is consistent with the results from the 2-Hz online TMS study from Zaretskaya et al. (2010), which also used individually defined target sites. The direction of our effect is opposite to the 1-Hz TMS study of Carmel et al. (2010) and the cTBS study of Kanai et al. (2011), both of which did not use individually defined targets. Arguably, the differences between the studies could, at least in part, be explained by the different localization methods. Indeed, a study using different localization methods revealed that localization based on individual fMRI coordinates is superior (in terms of power) to localization using standard group coordinates (Sack et al. 2009). The different localization methods could hence explain differences in effect sizes in our experiments (with Exp. 1 revealing an effect, but not so Exp. 2 and Exp. 3). However, we deem it unlikely that differences in target site localization could explain the inconsistent directions of the effects, with Exp. 1 and (Zaretskaya et al. 2010) showing a lengthening, while (Carmel et al. 2010, Kanai et al. 2011) reporting a shortening of percept durations, apart from the possibility of chance effects in the lower-powered studies.

### Experimental power

Was the power enough to detect an underlying effect following parietal TMS stimulation? Our experiments had the highest number of participants compared to previous parietal TMS studies on bistable perception (see Table 1). Correspondingly, we should have had sufficient power to detect the relatively large effect sizes reported in the cTBS studies we aimed to replicate (Kanai et al. 2010, 2011) (estimated effect size of >0.8, power: Exp. 1 = 0.92, Exp. 2 = 0.82, Exp. 3 = 0.91). Crucially, two of our three experiments led to no clear effect direction, and our first experiment, as well as Zaretskaya et al. (2010) and Kanai et al. (2010) led opposite effects (lengthenings), compared to Carmel et al. (2010) and Kanai et al. (2011) (shortenings). In line with these inconsistent results, our pooled analysis using combined data from the 41 unique participants revealed no general effect and the linear mixed models showed a weak lengthening only when viewing the SFM stimulus.

However, it is notable that the two cTBS experiments with shortening effects had the lowest number of participants, and that one of them (Carmel et al. 2010) had no vertex control condition. It can hence not be excluded that their findings constitute type I errors. Finally, as noted above, the possibility remains that individual functional localization makes a difference in both power and consistency of effect direction, which could account for the results in Exp. 1 and its consistency with another individually localizing online TMS study (Zaretskaya et al. 2010), and that of a high-powered TMS experiment on prefrontal regions that had similar fMRI responses as parietal cortex (Weilnhammer et al. 2021).

Despite this, the cTBS results are weak, and completely absent in non-individually localized experiments. On these grounds, we deem it unlikely for our repeated null results to be type II errors.

### Is cTBS effective over parietal cortex?

Lastly, we need to ask if cTBS is an effective method to consistently disrupt brain function in parietal cortex. Since the introduction of cTBS by Huang and colleagues in 2005 (Huang et al. 2005), cTBS has developed to be an established and validated inhibitory protocol for non-invasive brain stimulation. Recent meta-analysis not only revealed an inhibitory effect of motor cortex cTBS (Wischnewski and Schutter 2015, Chung et al. 2016), but also the existence of publication bias (Chung et al. 2016). Moreover, several studies have revealed weak to no effect following motor cortex cTBS. For example, a study investigating the effects of motor cortex cTBS in 420 participants revealed a large inter-subject variability with only ∼65% of subjects showing the expected MEP suppression after M1-cTBS (Corp et al. 2020) and in further well-powered motor cortex cTBS studies with a large sample size (*n* > 50) only ∼40% of the participants showed the expected inhibition (Hamada et al. 2013, McCalley et al. 2021).

Yet, in contrast to motor cortex cTBS studies, it is more difficult to assess the efficacy of *parietal* cTBS as we are lacking a direct physiological measure to quantify it (such as MEPs). The fact that the very same parietal cTBS protocol in the present study led to weak or no responses (and—in light of prior studies—to incongruent results) can be indicative that: (i) the parietal cortex is not involved in resolving perceptual ambiguity or (ii) that cTBS is ineffective over parietal cortex altogether. Our results cannot provide a conclusive answer as to which of these alternatives is correct.

If, like in motor cortex, a high inter-subject variability of cTBS effects accounts for these weak or null results, the following points can inform us:

First, if the variation of parietal cTBS effects observed is dependent on subject-dependent variables, such as genetic polymorphisms (Cheeran et al. 2008, Jannati et al. 2017, 2019) or the type of interneuron network recruited by TMS stimulation (Hamada et al. 2013), we hypothesized to see a correlation between motor-cortex cTBS-induced changes in MEP amplitude and the modulation of percept durations following parietal cTBS (Praß and de Haan 2019). This was not observed. Moreover, a subsequent analysis of the ‘responder’ group (*n *= 10) revealed no consistent effect following parietal cTBS stimulation.

Second, our behavioural findings of correlations between switch rates across subjects between distinct stimuli in Exp. 3 support the understanding that the bistable stimuli used share a common mechanism, making their comparison feasible. However, cTBS effects were uncorrelated between displays, opposite to the expectations if the variability by the observed cTBS effects were to be explained by subject-dependent variables.

In sum, our results indicate that *cTBS over the parietal cortex* is ineffectual in modulating perception during bistable viewing. Second, they suggest that this cannot be explained by individual variability measures that account for cTBS differences in motor cortex.

It is indeed possible that cTBS is ineffectual over parietal cortex in general, but our data support this generic conclusion only under the assumption that this area is indeed causally involved in modulating bistable viewing. We support this assumption not least due to our own prior causal evidence using a 2-Hz TMS study (Zaretskaya et al. 2010) and for additional reasons outlined in the next section.

We also note that the present data do not constitute an isolated failure to replicate stimulation reports using cTBS outside of the motor-cortex. Similar incongruent reports can be found in related visual consciousness research after stimulation of prefrontal, parietal, and occipital cortices. For example, while an early influential report revealed an impairment of metacognitive visual awareness following prefrontal cTBS (Rounis et al. 2010), subsequent comparable studies revealed an enhancement (Rahnev et al. 2016) or no effect at all (Bor et al. 2017). Also a recent parietal cTBS study (Praß and de Haan 2019) failed to replicate reports of TMS-induced extinction (Cazzoli et al. 2009). In occipital cortex, one cTBS study showed a decreased conscious detection and confidence of visual stimuli (Rahnev et al. 2013), while another report and replication thereof revealed an unexpected enhancement (Allen et al. 2014). Together, our results, the large inter-subject variability of cTBS effects and these further inconsistent cTBS results cast doubt on the general neural efficacy of cTBS and encourage caution when interpreting cTBS reports outside of motor cortex.

### Evidence for a causal parietal involvement in multistability

There is compelling evidence that supports the role of the frontoparietal cortex in resolving and modulating perceptual ambiguity. To begin with, the frontoparietal cortex has been consistently shown to modify perceptual dynamics via, for example, attentional mechanisms (Zhang et al. 2011, Li et al. 2017). In fact, correlational and causal evidence suggests that the frontoparietal cortex is indeed involved in resolving perceptual ambiguity: in addition to neuroimaging reports suggesting a frontoparietal involvement (Sterzer and Kleinschmidt 2007, Wang et al. 2013, Weilnhammer et al. 2013), two recent TMS experiments revealed a causal involvement of the frontal cortex in perceptual alternations (Watanabe 2021, Weilnhammer et al. 2021). The first TMS study used a close loop EEG-TMS approach to reveal a brain-state-dependent involvement of the prefrontal cortex in spontaneous switches of a SFM stimulus (Watanabe 2021). The second study applied cTBS over the prefrontal cortex and revealed reduced perceptual alternations (Weilnhammer et al. 2021). Interestingly, while this study applied cTBS only to prefrontal cortex to find slower perceptual alternations, the parietal fMRI responses in that study were similar to those of the targeted area of the prefrontal cortex (especially in the SPL). These results are congruent to our previous TMS results (using online 2 Hz TMS) on parietal cortex also showing a slowing of alternations (Zaretskaya et al. 2010).

Electrophysiological measurements on frontal areas of macaques reveal robust responses that correlate with and precede perceptual switches, even independent of behavioural responses (Panagiotaropoulos et al. 2012, Kapoor et al. 2022, Dwarakanath et al. 2023). And, although similar transient responses exist in posterior sensory cortices (de Jong et al. 2016), new evidence suggests that these could be the results from feedback from anterior higher-level non-sensory areas (Grassi et al. 2016a, de Jong et al. 2020).

It is important to note that the above evidence is also compatible with results from fMRI studies using no-report or invisible BR paradigms (Frässle et al. 2014, Brascamp et al. 2015, Zou et al. 2016). The reduced frontoparietal activity when participants do not report (Frässle et al. 2014) or perceive alternations (Brascamp et al. 2015, Zou et al. 2016) suggests a frontoparietal role in introspection and awareness. Nevertheless, the same studies show that several parieto-frontal regions remain involved in perceptual switches, supporting their possible causal role (Zaretskaya and Narinyan 2014, Weilnhammer et al. 2021).

### Synthesis and conclusion

Together, our results leave us to call into question the efficacy of cTBS over parietal cortex in affecting bistable perception, in particular as each of our experiments exceeded the experimental power of the matched prior cTBS studies. In this light, we would now like to turn our attention back to the original conundrum of inconsistent TMS results we aimed to resolve with these experiments. As our results cast doubt on prior cTBS studies, also the functional fractionation between IPS and pSPL (from Kanai et al. 2010, 2011) will have to be re-examined, as it is based on relatively weak cTBS evidence from small samples. Also, a single pulse TMS-EEG study revealed no systematic difference between IPS and SPL stimulation in neither behaviour nor evoked EEG signal (Schauer et al. 2016), and our prior online 2-Hz TMS study did not reveal any effect during SPL stimulation, while revealing a lengthening of percept durations when disrupting IPS (Zaretskaya et al. 2010). As shown in Table 1, the key inconsistency among non-cTBS studies remains between the lowest-powered study that did not include a vertex control and Zaretskaya et al. (2010). Note that our results leave undisputed the robust correlational evidence in support of a fractionation as revealed by the relationship between behaviour and grey-matter density in the parietal cortex (Kanai et al. 2010, 2011). These correlational results have been replicated for the anterior part of the right IPS (Sandberg et al. 2016) and have served as basis for the prediction of dominance durations based on fMRI-based energy landscape modelling (Watanabe et al. 2014), dynamic-causal-modelling (Megumi et al. 2014) and functional connectivity (Baker et al. 2015).

Altogether, as the series of inconsistent results cannot be explained by sample size (Exp. 1, 2, and 3), targeted areas (Exp. 2), stimuli used (Exp. 1 and 3), or inter-subject variability (Exp. 2 and 3), we tentatively conclude that parietal cTBS does not modulate bistable perception. Yet, in view of further conflicting evidence about cTBS effectiveness beyond the motor cortex, our cTBS results do not shed doubt on the parietal as well as frontal causal involvement in steering bistable perception. We suggest additional high-powered non-cTBS studies to resolve more fine-grained questions about parietal causal involvement in resolving perceptual ambiguity and possible functional fractionations therein.

## Supplementary Material

niae009_Supp

## Data Availability

Data are not publicly available but can be provided by the authors upon reasonable request.
